# Epstein-Barr Virus in the RIVERA Case-Control Study of Acute Febrile Illness: Acute Mononucleosis Nearly Absent as an Etiology in the Peruvian Amazon

**DOI:** 10.4269/ajtmh.24-0051

**Published:** 2024-11-12

**Authors:** Thomas G. Flynn, Maribel Paredes Olortegui, Paul F. Garcia Bardales, Francesca Schiaffino, Tackeshy N. Pinedo Vasquez, Wagner V. Shapiama López, Pablo Peñataro Yori, César J. Ramal Asayag, Graciela R. Meza Sánchez, Josh M. Colston, Margaret N. Kosek

**Affiliations:** ^1^ Division of Infectious Diseases and International Health, School of Medicine, University of Virginia, Charlottesville, Virginia;; ^2^ Asociación Benéfica Prisma, Investigaciones Biomédicas, Iquitos, Perú;; ^3^ Faculty of Veterinary Medicine, Universidad Peruana Cayetano Heredia, San Martín de Porres, Perú;; ^4^ Faculty of Medicine, Universidad Nacional de la Amazonía Peruana, Iquitos, Perú

## Abstract

Large diagnostic panels allow for pathogens with high or low likelihood of causing attributable illness to be tested simultaneously. Infectious mononucleosis (IM) due to primary infection with Epstein-Barr virus (EBV) is a common cause of acute febrile illness (AFI) in case series from high-income countries, though its contribution to AFI in tropical low-income settings is unclear. As part of a case-control study using multiplex quantitative polymerase chain reaction (qPCR) diagnostics, we set out to determine if primary EBV infection was an underrecognized cause of AFI in the Peruvian Amazon. Presence of EBV DNA in whole-blood samples was equally prevalent among febrile cases and afebrile controls (34.6% [247/714] versus 35.7% [248/695]) and was not correlated with classic IM symptoms. Given the clear lack of clinical significance of the whole-blood PCR results, additional testing was pursued to ascertain the true prevalence of IM among cases of AFI in this population. The presence of EBV DNA in plasma, a marker of active EBV-related processes, was detected in 7% (5/68). Anti–EBNA-1 IgG, a late marker of prior infection, was tested via ELISA and detected in 4/5 of the plasma-positive patients, thereby excluding an acute primary EBV infection in all but one patient. Infectious mononucleosis due to primary infection with EBV was not an important etiology of AFI in the Peruvian Amazon, despite high rates of initial test positivity.

## INTRODUCTION

The accurate diagnosis of acute febrile illness (AFI) poses a unique challenge in tropical regions where multiple endemic diseases coexist, and nonspecific or overlapping symptoms make differentiation impossible when based on clinical features alone.^1^ Furthermore, a lack of adequate standardized diagnostic methods makes interpretation of testing difficult and hinders population-level surveillance.^2^ This is true for well-known causes of AFI, such as infectious mononucleosis (IM) due to primary infection with Epstein-Barr virus (EBV), for which a combination of serology, molecular testing, and clinical context may all be required for accurate diagnosis.^3^^–^^6^

Human gammaherpesvirus 4, known commonly as EBV, is notable for its ubiquity, its role in causing IM upon primary infection, and the establishment of lifelong latency within B lymphocytes.^7^^,^^8^ It has an etiologic role in the range of pathologic processes, having been associated with endemic Burkitt’s lymphoma and a range of B- and T-cell malignancies.^9^^–^^11^

Previous studies of EBV’s role in AFI in the tropics are limited to descriptive cohorts and case series. Serologic studies of AFI etiology in Tanzania and India have attributed 1–4% of cases to primary infection with EBV,^12^^,^^13^ whereas molecular studies using whole blood in Kenya^14^ and plasma in Tanzania^15^ detected EBV DNA in samples from 27% and 45% of patients, respectively.

In the Amazonian region of northeastern Perú, year-round active surveillance of AFI is ongoing using testing for a panel of endemic pathogens as part of RIVERA,^16^ a prospective health facility–based case-control study. Whole-blood samples from patients with AFI and their age-, sex-, and location-matched controls were assessed using a modular array of quantitative real-time polymerase chain reaction (qRT-PCR) primers for 26 pathogens of interest.^17^^,^^18^ Epstein-Barr virus DNA was the most common target detected among cases but was equally prevalent among controls and was therefore not associated with febrile illness. This finding prompted reassessment of the natural history of EBV infection and the clinical interpretation of EBV molecular testing results.

Although low levels of detectable EBV DNA are known to persist for life in immunocompetent individuals, this is thought to be restricted to the intracellular compartment, where one in every 104–106 circulating memory B lymphocytes harbors one or two copies of the viral genome in the form of episomal DNA.^19^^,^^20^ Unlike detection in whole-blood samples, however, extracellular or cell-free EBV DNA in plasma samples has previously been reported to be undetectable among healthy immunocompetent hosts and is highly specific for acute EBV-related illnesses such as primary infection, reactivation, or malignancy.^21^^,^^22^ We therefore hypothesized that detection of EBV DNA in whole-blood samples for at least some participants was likely due to latent intracellular infection, with EBV DNA absent from the extracellular compartment.

To distinguish between evidence of active EBV-related processes (which could be implicated in febrile illness) and mere intracellular latency, we repeated EBV DNA testing using plasma samples for a subset of the participants whose whole blood had been positive for EBV DNA. To identify primary infection, which is the EBV-related process most likely to be associated with fever, we also assessed the presence of antibodies against EBV nuclear antigen type-1 (anti-EBNA-1 IgG), a marker of remote prior exposure to latent-phase EBV infection, which only becomes detectable 5 weeks or more after initial infection. The combined findings of the three test results allowed for categorization of participants by active versus latent infection and previously exposed versus unexposed, thereby identifying possible instances of AFI due to primary EBV infection.

## MATERIALS AND METHODS

**Figure 1.**
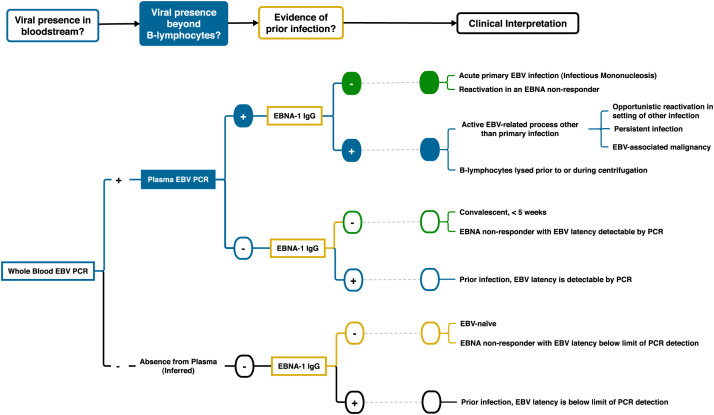
Systematic approach to clinical interpretations of combined results from qPCR testing for Epstein-Barr viral DNA in whole blood, in plasma, and from anti–EBNA-1 IgG serologies by ELISA. Although traditionally diagnosed by heterophile antibodies, patients with acute symptomatic primary Epstein-Barr virus infection, or infectious mononucleosis (IM), will have not only detectable viral DNA in whole blood (blue) but also detectable cell-free viral DNA in plasma samples (solid fill), as well as no serologic evidence of remote prior infection when testing for immunity to EBNA (yellow), a viral protein only expressed during latency, which takes 5 weeks before an IgG response is consistently present. This allows an iterative approach to excluding IM in febrile patients who initially test positive for EBV DNA in whole blood via PCR (only the solid green category could be consistent with IM), as multiple other clinical scenarios, including the typical lifelong intracellular latency after remote infection, may also result in whole-blood viral detection. EBNA-1 = EBV nuclear antigen type-1; EBV = Epstein-Barr virus; PCR = polymerase chain reaction; qPCR = quantitative PCR.

The systematic approach to identifying possible primary EBV infections is outlined in Figure 1.

### Sample selection.

**Figure 2.**
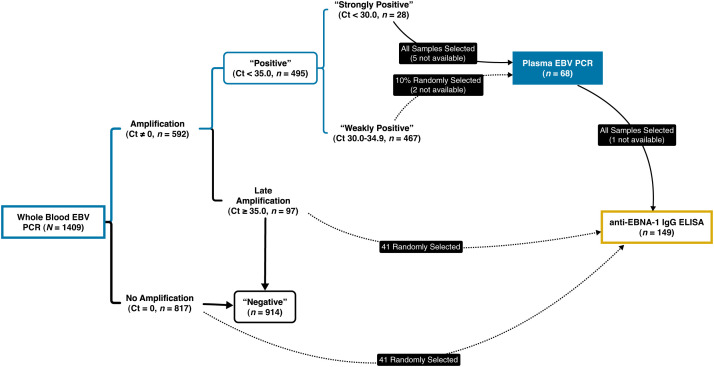
Sampling strategy for plasma EBV PCR and anti–EBNA-1 IgG ELISA based on initial whole-blood EBV PCR results. Whole blood samples (*N* = 1,409) were tested for EBV DNA as part of a multiplex qPCR panel for pathogens suspected of causing acute febrile illness. Those that failed to amplify (*n* = 817) as well as those that showed late amplification at cycle 35 or beyond (*n* = 97) were considered negative (*n* = 914), whereas those that exhibited amplification prior to cycle 35 were considered positive (*n* = 495). For follow-up PCR testing in plasma, those that had amplified prior to cycle 30 (“Strongly Positive”; *n* = 28) were prioritized for follow-up PCR testing in plasma, whereas 10% of the remaining “Weakly Positive” (*n* = 467) group were randomly selected for investigation. All samples that underwent plasma PCR testing, as well as subsets of whole blood–negative patients, were then tested via ELISA for anti–EBNA-1 IgG to assess serologic evidence of prior infection with EBV. Ct = cycle threshold; EBNA-1 = EBV nuclear antigen type-1; EBV = Epstein-Barr virus; qPCR = quantitative polymerase chain reaction.

Patients from the city and environs of Iquitos, Maynas Province, Loreto Department, Perú, who were previously enrolled in a surveillance study^16^ of AFI and their age, sex, and location-matched afebrile controls (*N* = 1,409) had previously been tested via TaqMan quantitative PCR (qPCR) Array Card (TAC)^17^ for the presence of EBV and 25 other organisms in their whole-blood samples (Figure 2). A cycle threshold (Ct) of less than 35 was considered positive, with a Ct of 35 or greater interpreted as negative.

In an effort to prioritize the analysis of subjects most likely to have active EBV-related disease, febrile case and afebrile control participants whose whole blood had been positive for EBV DNA were further classified into the following subcategories: “Strongly Positive” (Ct <30.0), from which all subjects were selected for further analysis, as well as “Weakly Positive” (35.0 > Ct ≥ 30.0), from which a random 10% subset was selected (Figure 2). Selection was without regard to febrile illness status, and initial case-control pairing was not maintained. Available plasma samples from these subjects, from same enrollment-day collection as the whole-blood samples, were tested for EBV DNA using qPCR.

### EBV DNA detection via qPCR.

Blood was drawn via venipuncture into ethylenediaminetetraacetic acid (EDTA) tubes and refrigerated prior to centrifugation and separation within 6 hours of specimen collection. Plasma was then stored at −80°C until time of analysis. Plasma samples were thawed prior to extraction of nucleic acid using the High Pure Viral Nucleic Acid Large Volume Kit (F. Hoffmann-La Roche Ltd, Basel, Switzerland). Extractions were performed with 4 mL of plasma or whole blood. A 18 *µ*M:5 *µ*M primer-probe mix was prepared with the same primers used in the initial assay, which were specific for a 90-bp fragment of EBV polymerase BALF-5 (forward 5′-CGGAAGCCCTCTGGACTTC-3′, reverse 5′-CCCTGTTTATCCGATGGAATG-3′, Probe FAM-TGTACACGCACGAGAAATGCG-MGB).^23^^–^^25^

One microliter of each extracted sample was combined with 2.5 *µ*L of TaqMan^™^ Fast Virus 1-Step Mastermix (Applied Biosystems, Thermo Fisher Scientific Inc., Waltham, MA), 0.5 *µ*L of primer-probe mix, and 6 *µ*L of nuclease-free H_2_O, for a total of 10 *µ*L in each reaction well. The reaction plate was then sealed and centrifuged at 500 *g* for 1 minute prior to thermocycling. Thermocycling involved initial denaturation and Taq polymerase activation at 95°C for 20 seconds, followed by amplification and target detection conducted over 40 cycles, with each cycle consisting of 95°C for 3 seconds, then 60°C for 30 seconds, followed by a plate read prior to the next cycle.

A lyophilized positive control consisting of a plasmid containing target sequences for all DNA targets tested by TAC^26^^,^^27^ was obtained from GENEWIZ (Azenta Life Sciences, South Plainfield, NJ) and was reconstituted in 300 *µ*L of standard diluent to yield a stock solution of concentration $0.87*10^{6}$ copies/*µ*L. The solution was serially diluted four times by 10-fold to generate a standard curve, allowing correlation between PCR Ct and EBV DNA copy concentration, with final values expressed as log_10_(copies EBV DNA/mL plasma):

$$\log_{10}\left(\text{copies}/mL\right)=\log_{10}\left(1000*10^{\left(\frac{Ct-43.156}{-3.29}\right)}\right)$$

Given that all participants for whom plasma was tested were already known to have detectable EBV DNA in their whole blood at Ct <35 (>5.48 log_10_(copies EBV DNA/mL whole blood)), any amplification, regardless of Ct, was considered a positive result in the plasma PCR assay.

### Anti–EBNA-1 IgG serology.

Anti–EBNA-1 IgG ELISA was performed on the same samples that had been subjected to qPCR. In addition, participants whose whole blood had initially tested negative by PCR were tested for the presence of anti–EBNA-1 IgG to determine whether they had a history of EBV infection. Because it was unknown whether a negative result due to no amplification and a negative result due to amplification at a high Ct can represent distinct clinical phenotypes, an equal number (*n* = 41) of participants was randomly selected from each of these subgroups (Figure 2). To assess the predominant age of EBV exposure in the study population, sera from children aged 8–26 months (*n* = 29) as well as from control subjects aged 10–15, 15–20, 20–25, and 25–30 years were randomly selected for analysis (*n* = 56; *n* = 14 from each age range).

Enzyme-linked immunosorbent assay kits using recombinant EBNA-1 for the detection of anti–EBNA-1 IgG antibodies were obtained from Immuno-Biological Laboratories, Inc. (IBL-America, Minneapolis, MN). Samples as well as positive, negative, and cutoff controls were analyzed per manufacturer’s protocol with results expressed in ELISA units (U).

## STATISTICAL ANALYSES

All analyses were conducted using R v. 4.3.2^28^ in RStudio using the Tidyverse^29^ and gtsummary^30^ packages.

Association of whole-blood EBV positivity with participant characteristics was assessed using univariate logistic regression, with each characteristic serving as the sole predictor and whole blood result as the binary outcome, with results expressed as odds ratios and 95% CIs (Table 1).

**Table 1:** Predictors of the presence of EBV DNA in whole blood by PCR

Predictor	OR (95% CI)*	*P*-Value
Demographic Information		
Age (years)	1.01 (1.00–1.02)	**0.003**
Male Sex	1.09 (0.87–1.37)	0.45
Household Income (soles/month)	1.00 (1.00–1.00)	0.94
BMI (kg/m^2^)	1.00 (0.98–1.02)	0.98
Crowding (occupants/bedroom)	1.11 (1.02–1.20)	**0.016**
Clinical Symptoms		
Sore Throat	1.25 (0.96–1.64)	0.10
Fatigue	1.09 (0.85–1.39)	0.51
Myalgia	1.15 (0.91–1.46)	0.23
Headache	1.02 (0.82–1.28)	0.85
Abdominal Pain	0.84 (0.63–1.12)	0.24
Nausea	0.98 (0.76–1.26)	0.86
Emesis	1.14 (0.79–1.66)	0.50
Clinical Signs		
Fever (Case)	0.95 (0.77–1.19)	0.67
Lymphadenopathy	1.94 (0.47–13.0)	0.41
Jaundice	1.02 (0.41–2.73)	0.97
Medical History		
History of HIV	0.18 (0.01–1.42)	0.14
Personal History of Cancer	1.65 (0.21–33.4)	0.67
History of Tuberculosis	0.68 (0.18–2.77)	0.57
Intensive Care	0.82 (0.14–6.25)	0.83
History of COVID-19 Diagnosis	1.05 (0.69–1.63)	0.83
COVID-19 Requiring Hospitalization	0.62 (0.22–1.77)	0.36
COVID-19 Requiring Intensive Care	0.27 (0.04–1.39)	0.13

BMI = body mass index; COVID-19 = coronavirus disease 2019; EBV = Epstein-Barr virus; OR = odds ratio; PCR = polymerase chain reaction. Bolded values significant at *α* = 0.05.

* CI calculated by univariate logistic regression.

Association of plasma EBV positivity with participant characteristics was assessed via Fisher’s exact test for categorical variables and via Wilcoxon rank-sum test for continuous variables. Multiple comparisons *P*-value correction was not performed.

Association of EBNA seropositivity with age was assessed using univariate logistic regression, with age serving as the sole predictor and the EBNA result as the binary outcome.

## RESULTS

### Participant characteristics and whole-blood positivity.

Whole-blood samples from a total of 1,409 participants had been previously tested for EBV DNA (Figure 1). Participants who were positive (*n* = 495) and those who were negative (*n* = 914) for EBV DNA in whole blood did not differ with respect to case versus control status nor with respect to symptoms commonly associated with infectious mononucleosis, such as sore throat, lymphadenopathy, and abdominal pain (Table 1). Each additional year of age and each additional occupant per bedroom in a household were associated with 1.01 (95% CI: 1.00–1.02) and 1.11 (95% CI: 1.02–1.20) times greater odds of whole-blood positivity, respectively (Table 1). Among healthy controls testing positive in whole blood (*n* = 248), viral loads ranged from 5.48 to 7.87, with mean of 6.01 and median of 5.91 log_10_(copies/mL).

### Epstein-Barr virus PCR in plasma.

Of the 68 patients whose whole blood had initially tested positive for EBV DNA in whole blood and who were selected for repeat qPCR testing in plasma, only five (7.4%) were positive for EBV DNA in plasma (Tables 2 and 3). The remaining 92.6% (63/68) harbored only intracellular EBV DNA, as is expected with latent infection of B lymphocytes. Plasma-positive subjects had higher mean whole-blood loads compared with the mean whole-blood loads of plasma-negative subjects (7.57 versus 6.31, *P* = 0.002, Wilcoxon rank-sum; Table 2). Four of the five plasma-positive samples were from febrile cases, though the association between plasma positivity and febrile illness status did not reach significance. Age, sex, and typical infectious mononucleosis symptoms were not associated with plasma positivity.

**Table 2:** Characteristics of subjects testing positive or negative for plasma EBV DNA by PCR

Predictor	Plasma EBV DNA (*N* = 68)		*P*-Value*
	Positive	Negative	
	*n* = 5	*n* = 63	
Demographic Information			
Age (years), Mean (SD)	30.00 (17.03)	36.54 (15.09)	0.29
Male Sex, *n* (%)	4 (80)	26 (41)	0.16
BMI (kg/m^2^), Mean (SD)	22.97 (4.79)	26.69 (4.55)	0.12
Household Income (soles/month), Mean (SD)	1,043.33 (905.34)	1,178.43 (1,191.26)	0.96
Crowding (occupants/bedroom), Mean (SD)	1.67 (1.15)	2.00 (1.06)	0.47
Clinical Symptoms, *n* (%)			
Sore Throat	1 (20)	15 (24)	>0.99
Fatigue	3 (60)	22 (35)	0.35
Myalgia	2 (40)	23 (37)	>0.99
Headache	1 (20)	29 (46)	0.37
Abdominal Pain	0 (0)	12 (19)	0.58
Nausea	2 (40)	19 (30)	0.64
Emesis	2 (40)	7 (11)	0.13
Clinical Signs			
Fever (Case), *n* (%)	4 (80)	35 (56)	0.38
Whole Blood EBV DNA (log_10_[copies/mL]), Mean (SD)	7.57 (0.48)	6.31 (0.70)	**0.002**
Lymphadenopathy, *n* (%)	0 (0)	0 (0)	–
Jaundice, *n* (%)	0 (0)	2 (3.2)	>0.99
Medical History, *n* (%)			
History of HIV	1 (20)	0 (0)	0.074
History of COVID-19 Diagnosis	0 (0)	7 (11)	>0.99
Personal History of Cancer	0 (0)	0 (0)	–
History of Tuberculosis	0 (0)	0 (0)	–
Intensive Care	0 (0)	0 (0)	–
COVID-19 Requiring Hospitalization	0 (0)	0 (0)	–
COVID-19 Requiring Intensive Care	0 (0)	0 (0)	–

BMI = body mass index; COVID-19 = coronavirus disease 2019; EBV = Epstein-Barr virus; PCR = polymerase chain reaction. Bolded values significant at *α* = 0.05.

* Comparisons of categorical variables via Fisher’s exact test, continuous variables via Wilcoxon rank-sum test.

**Table 3:** Clinical characteristics of participants positive for EBV DNA in plasma by PCR

Febrile?	Whole-Blood EBV (log_10_ copies DNA/mL)	Plasma	Anti-EBNA-1 IgG	Age (years), Sex	Symptoms	Presentation	Codetection
Yes	7.298	Positive	Negative	34, Male	Fever, Dry Cough, Chest Pain, Fatigue, Myalgia, Nausea	Stable Vital Signs	*Plasmodium* spp.
Yes	7.211	Positive	Positive	58, Male	Fatigue, Headache, Nausea and Vomiting	Hypotensive and ∼Hypoxic	CMV
Yes	8.383	Positive	Positive	22, Male	Prod. Cough, Sore Throat, Fatigue, Emesis, Diarrhea	History of Renal Disease, Tachycardic to 153	CMV
Yes	7.595	Positive	Positive	20, Female	Fever, Dry Cough, Myalgia	Pregnant	*Plasmodium* spp.
No	7.354	Positive	Positive	16, Male	n/a	History of HIV	*Plasmodium* spp.

CMV = cytomegalovirus; EBNA-1 = EBV nuclear antigen type-1; EBV = Epstein-Barr virus; n/a = not applicable; PCR = polymerase chain reaction.

### Anti–EBNA-1 IgG serology.

**Figure 3.**
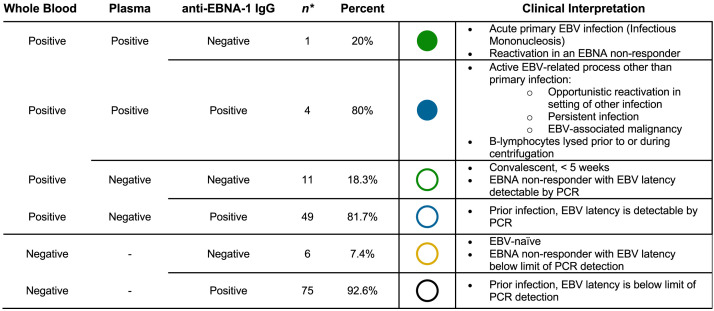
Prevalence and clinical interpretation of combined molecular and serologic results. *One whole blood–negative patient and two whole blood–positive/plasma-negative patients had equivocal ELISA results and were excluded from further analysis. EBNA-1 = EBV nuclear antigen type-1; EBV = Epstein-Barr virus; PCR = polymerase chain reaction.

Only one of the five plasma-positive subjects was negative for anti–EBNA-1 IgG and therefore potentially consistent with primary EBV infection (Figure 3). Clinical presentations of plasma-positive participants are described in Table 3. The majority (81.7%, 49/60) of whole blood–positive and plasma-negative participants were anti-EBNA positive, suggesting prior infection and current persistence of intracellular viral DNA only, consistent with healthy latency (Figure 3). Similarly, 92.6% of whole blood–negative participants were also anti-EBNA positive, again suggesting prior infection, though with latent intracellular viral DNA being below the limit of PCR detection (Figure 3).

### Seroprevalence and age.

**Figure 4.**
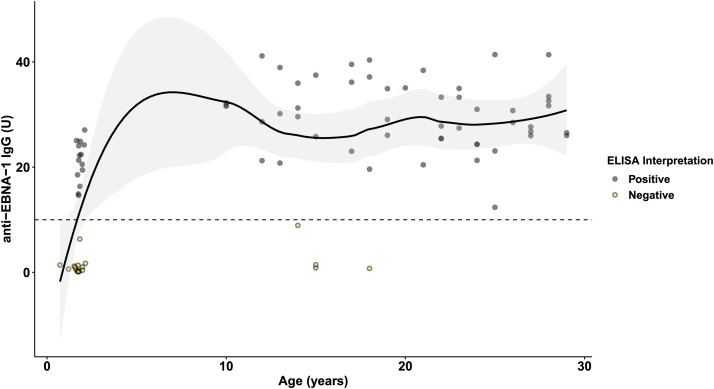
Serologic evidence of prior EBV exposure by age. Seropositivity with IgG antibodies against EBNA-1 increased markedly in the first 2 years of life in the population, as has been described in other low- and middle-income settings. It is thought that early exposure to EBV results in a pauci- or asymptomatic primary infection, whereas exposure and primary infection during adolescence often leads to the febrile illness known as infectious mononucleosis. These aspects of local epidemiology and EBV natural history likely explain why infectious mononucleosis is essentially absent as an etiology of acute febrile illness in this population. *n* = 85, curve is LOESS fit with standard error range. Dashed line represents positivity threshold. EBNA-1 = EBV nuclear antigen type-1; EBV = Epstein-Barr virus.

The age–seroprevalence curve suggests that exposure to EBV occurs early in life for the majority of the population (Figure 4). Each additional year of age was associated with 1.18 times greater odds of EBNA positivity (95% CI: 1.10–1.30, *P* <0.001).

## DISCUSSION

Critical examination of cases of AFI in the Peruvian Amazon that initially tested positive for EBV DNA by multiplex PCR using whole blood revealed that few, if any, were compatible with an active EBV-related etiology. The presence of intracellular latent viral DNA is the most plausible explanation for the frequent detection of EBV DNA in whole-blood samples among this cohort.

Our findings suggest that most individuals in this population have previously been exposed to EBV and that this exposure most often occurs in early childhood, consistent with prevailing theories regarding an early age of asymptomatic EBV acquisition in low- and middle-income countries.^20^^,^^23^^,^^31^^,^^32^ Given the lifelong nature of infection, the detection of EBV DNA in the whole blood of only one-third of participants is therefore likely a function of both the limit of detection of our qPCR assay and the average latent viral “set point” of this population,^33^ with whole blood-negative/EBNA-positive individuals actually latently infected, but with a set point that is below the limit of detection. Although the distribution of viral loads in whole blood among healthy controls is left-censored at our limit of detection of 5.49 log_10_(copies/mL) and neither we nor prior authors have taken steps to impute values for non-detects, our mean, median, and upper-bound values are among the highest reported in the literature for healthy control subjects.^22^^,^^34^^–^^38^ Immunosenescence raising the latent set point above the detection threshold likely explains the subtle association we observed between increasing age and increasing odds of latent viral detection.^33^

Accordingly, we observed a very low proportion of whole blood–positive participants who were also positive for cell-free EBV DNA in plasma, again suggesting that the majority of subjects did not have an active EBV-related process at the time of sampling. As for the few plasma-positive individuals, the extracellular presence of EBV DNA is unlikely to have been artefactual, as lysis of lymphocytes during handling of certain samples would have been a stochastic occurrence. Instead, EBV DNA presence in plasma was clearly associated with higher total levels of EBV DNA in whole blood, suggesting an increased viral presence beyond the background intracellular latency.

Although primary infection is traditionally diagnosed with nonspecific heterophile antibodies as well as EBV-specific antibodies against the viral capsid antigen, qPCR also appears useful for diagnosis of acute or primary infections,^21^^,^^23^^,^^39^^,^^40^ the rationale for its inclusion in the panel of AFI pathogens tested by the parent study. Acute primary infection with EBV as a cause of fever was further excluded by positive serologic testing for anti–EBNA-1 IgG for all but one plasma-positive participant whose initial TAC testing had also detected *Plasmodium* spp., making malaria a more clinically likely etiology.

There remains a range of possible explanations for EBV presence in plasma among the four EBNA+ subjects. An opportunistic reactivation of EBV in the context of severe illness, systemic inflammation, or infection with other pathogens has been described.^41^^,^^42^ In addition, various EBV-related malignancies, which are noted to be relatively prevalent in Peruvian populations relative to neighboring Latin American countries,^9^ may also effect a detectable level of EBV DNA in plasma. Molecular detection of EBV DNA using qRT-PCR has been applied to screening, diagnosis, and monitoring of EBV-associated malignancies and post-transplant lymphoproliferative disorder.^6^^,^^19^^,^^43^

Our finding of no AFI cases attributable to EBV infection contrasts sharply with that of previous studies, which relied on serologies^12^^,^^13^ or molecular testing in whole blood only^14^ or lacked matched afebrile control subjects for comparison,^15^ and likely overestimated the actual contribution of EBV infection to AFI. Although a similar study using TAC for AFI pathogen detection in Tanzania also noted a markedly lower EBV detection rate in plasma compared with whole blood, the authors attributed this finding to a difference in sensitivity between qPCR and metagenomic next-generation sequencing modalities.^15^ Our similar finding of plasma–whole blood discordance, this time using the single-plex version of the same TaqMan PCR assay, suggests that this phenomenon is best explained by a difference in sample type.

Limitations of this investigation include retesting only a subset of whole blood–positive subjects for plasma EBV DNA. However, the prioritization of higher whole-blood levels for plasma testing and the finding of plasma positivity at only the highest range of that subset suggest that our analysis likely captured all plasma-positive subjects. Our assay was also not calibrated to the WHO’s standard, which limits quantitative comparison of our results with those of other investigators; however, as our goal was internal comparability, standardization against the same proprietary positive control that had already been used in the multiplex whole blood assay was prioritized.

In summary, case-control designs are critically important to the study of AFI, especially when utilizing highly sensitive culture-independent methods of pathogen detection. Careful consideration should be given to the sample type used in multiplex molecular testing to reduce diagnostic ambiguity, and reflex to ancillary tests may be required to further refine the clinical interpretation of an initial positive result. The use of whole-blood samples in multiplex qPCR assays for AFI surveillance and attribution, while enhancing sensitivity of detection for some intracellular pathogens such as *Plasmodium* spp. and *Rickettsia* spp.,^17^ may confound interpretation of others such as EBV, which exhibit a latent intracellular presence. Future studies evaluating EBV as a potential etiology of AFI should not rely on PCR testing of whole blood alone. Alternative strategies for improved diagnostics would include PCR testing of plasma as either the initial modality of choice or as reflex testing for confirmation of possible acute EBV-related illness, ideally accompanied by serologic testing to aid clinical interpretation.
